# Two Uncomplicated Vaginal Deliveries in a Woman With Scimitar Syndrome: A Case Report

**DOI:** 10.7759/cureus.104674

**Published:** 2026-03-04

**Authors:** Motoki Tanabe, Toshiyuki Itai, Chika Akamatsu, Shinya Kondo, Rie Nakashima, Shun Kawai, Yuichi Imai, Etsuko Miyagi

**Affiliations:** 1 Department of Obstetrics and Gynecology, Yokohama City University School of Medicine, Yokohama, JPN; 2 Department of Cardiology, Yokohama City University School of Medicine, Yokohama, JPN; 3 Department of Pediatric Cardiology, Yokohama City University School of Medicine, Yokohama, JPN

**Keywords:** adult congenital heart disease (achd), adult scimitar syndrome, multidisciplinary care approach, normal vaginal delivery, obstetric epidural, partial anomalous pulmonary venous return (papvr)

## Abstract

Scimitar syndrome is a rare, complex congenital cardiopulmonary venous anomaly characterized by anomalous drainage of the right pulmonary vein into the inferior vena cava, hypoplasia of the right lung, and dextroposition of the heart. Only a few reports mentioned perinatal outcomes in women with scimitar syndrome. We report two uncomplicated deliveries in a woman with scimitar syndrome, which was diagnosed and surgically palliated at 15 and 19 years of age, respectively. Having had dyspnea on exertion (New York Heart Association (NYHA) Class II), recurrent pneumonia, and hemoptysis, she underwent coil embolization of a systemic artery (renal artery) to the right lung and video-assisted thoracoscopic ligation of the scimitar vein at 19 years of age, which ameliorated her dyspnea on exertion (NYHA Class I). After her cardiac function was confirmed normal without pulmonary hypertension by echocardiography, she was allowed to plan a pregnancy and conceived spontaneously at 23 and 26 years of age. During these pregnancies, she underwent serial echocardiography tests, which showed her cardiac function was stable without pulmonary hypertension. She achieved uncomplicated vaginal deliveries with epidural anesthesia at term. This clinical experience highlights the importance of a multidisciplinary approach for pregnancy and delivery in women with scimitar syndrome. It also demonstrates that favorable maternal and neonatal outcomes are possible in women with surgically palliated scimitar syndrome by ligating the scimitar vein when cardiac function is stable and pulmonary hypertension is denied.

## Introduction

Scimitar syndrome is a rare, complex congenital cardiopulmonary venous anomaly characterized by anomalous drainage of the right pulmonary vein into the inferior vena cava, right lung hypoplasia, and dextroposition of the heart [1]. An estimated incidence of scimitar syndrome is one to three per 100,000 live births, with a female predominance, although the estimation could be underestimated due to delayed diagnosis in adulthood for milder patients or asymptomatic patients [2]. The term ‘scimitar’ refers to a crescent-shaped sword, describing the characteristic appearance of the anomalous pulmonary vein on chest radiography. Its disease severity varies, depending on the age of onset and associated congenital heart defects; exemplary cardiac defects associated with scimitar syndrome include atrial septal defect, ventricular septal defect, patent ductus arteriosus, and pulmonary vein stenosis [2]. These complications can influence surgical decision-making; they can also lead to congestive heart failure and pulmonary hypertension, with which pregnancies are contraindicated [3,4,5]. Surgical repair, especially when performed early in life, improves prognosis, and the overall survival rate in adulthood is estimated at over 85% [1].

Although several hundred patients with scimitar syndrome have been reported, only a handful of reports mentioned pregnancy outcomes in women with scimitar syndrome, none of whom were diagnosed with pulmonary hypertension, but the majority delivered their babies by caesarean section [6-11]. Further reports are warranted to delineate the clinical profile of pregnancies in women with scimitar syndrome.

Here, we report two uncomplicated vaginal deliveries in a woman with scimitar syndrome without associated intracardiac lesions, who had coil embolization of an anomalous systemic artery from the right renal artery to the right lung and video-assisted thoracoscopic ligation of the scimitar vein at 19 years old, with no complications thereafter. This clinical experience demonstrates that favorable maternal and neonatal outcomes are possible in women with palliated scimitar syndrome when cardiac function is stable and highlights the importance of careful follow-up of patients with scimitar syndrome, even if they do not have cardiac defects.

## Case presentation

The patient was a 26-year-old woman, gravida 2 para 1. She had one uneventful vaginal delivery at our institution and visited us for another pregnancy management after conceiving spontaneously. She took a selective serotonin reuptake inhibitor for anxiety disorder. Her mother was on medication for hypertension, and her father was diagnosed with diabetes mellitus. Her relatives were not diagnosed with heart disease.

**Figure 1:**
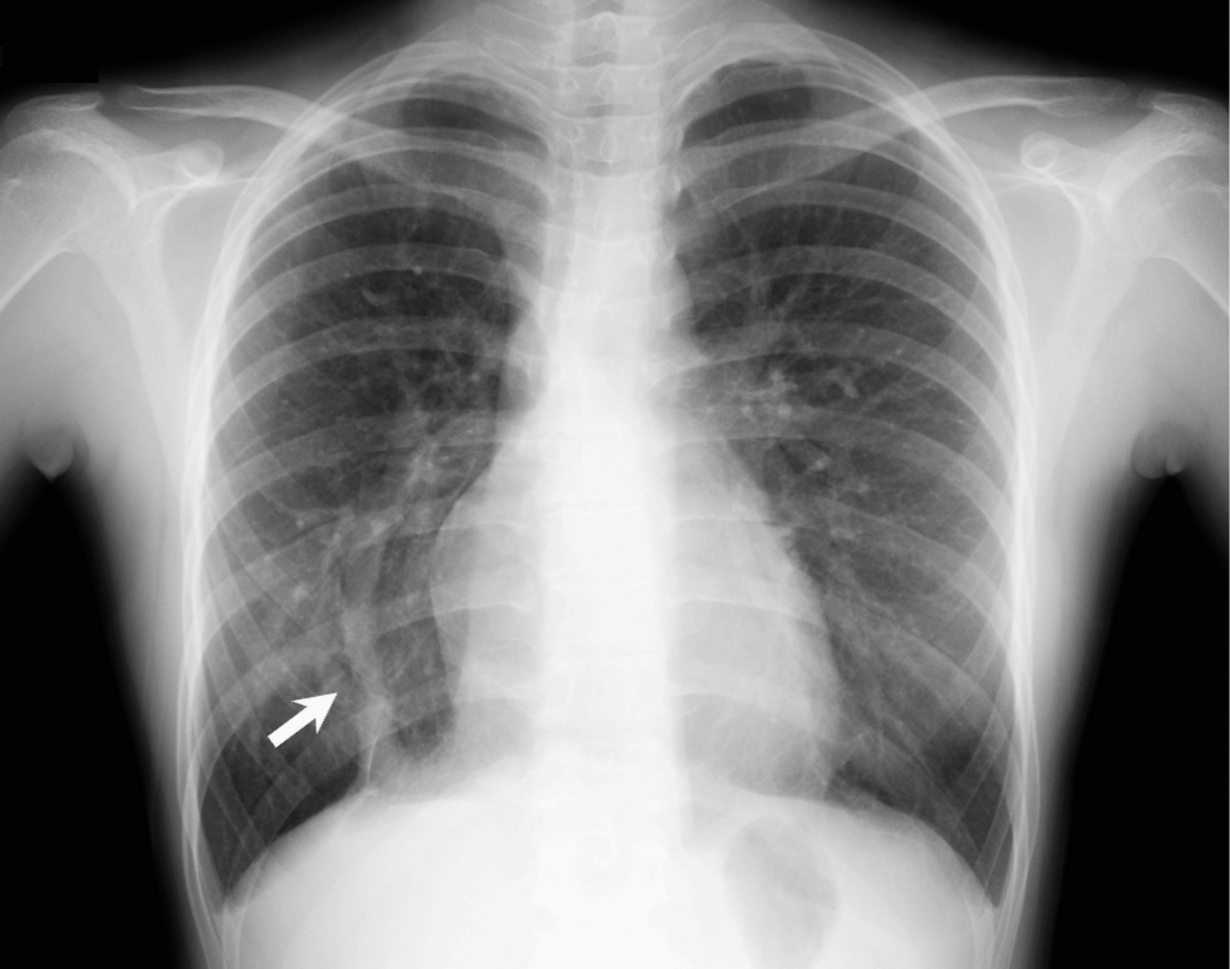
Chest X-ray at 15 years showing the scimitar vein in the right lung (arrow).

The patient experienced easy fatigability in childhood, although no medical evaluation was undertaken. An electrocardiogram at a school medical checkup revealed right bundle branch block at 12 years of age, with no further follow-up. At 15 years of age, she was diagnosed with scimitar syndrome by a chest X-ray (Figure 1) and subsequent computed tomography (CT) scans. She had an anomalous pulmonary venous from the right lung to the inferior vena cava and an anomalous vessel from the right renal artery to the right lung. There were no associated intracardiac lesions. Although she had dyspnea on exertion, invasive treatment was not pursued because echocardiography demonstrated a low shunt volume, with an estimated Qp/Qs of 1.2 (< 1.5: considered a hemodynamically insignificant shunt). She was instead advised to avoid intense exercise.

**Figure 2:**
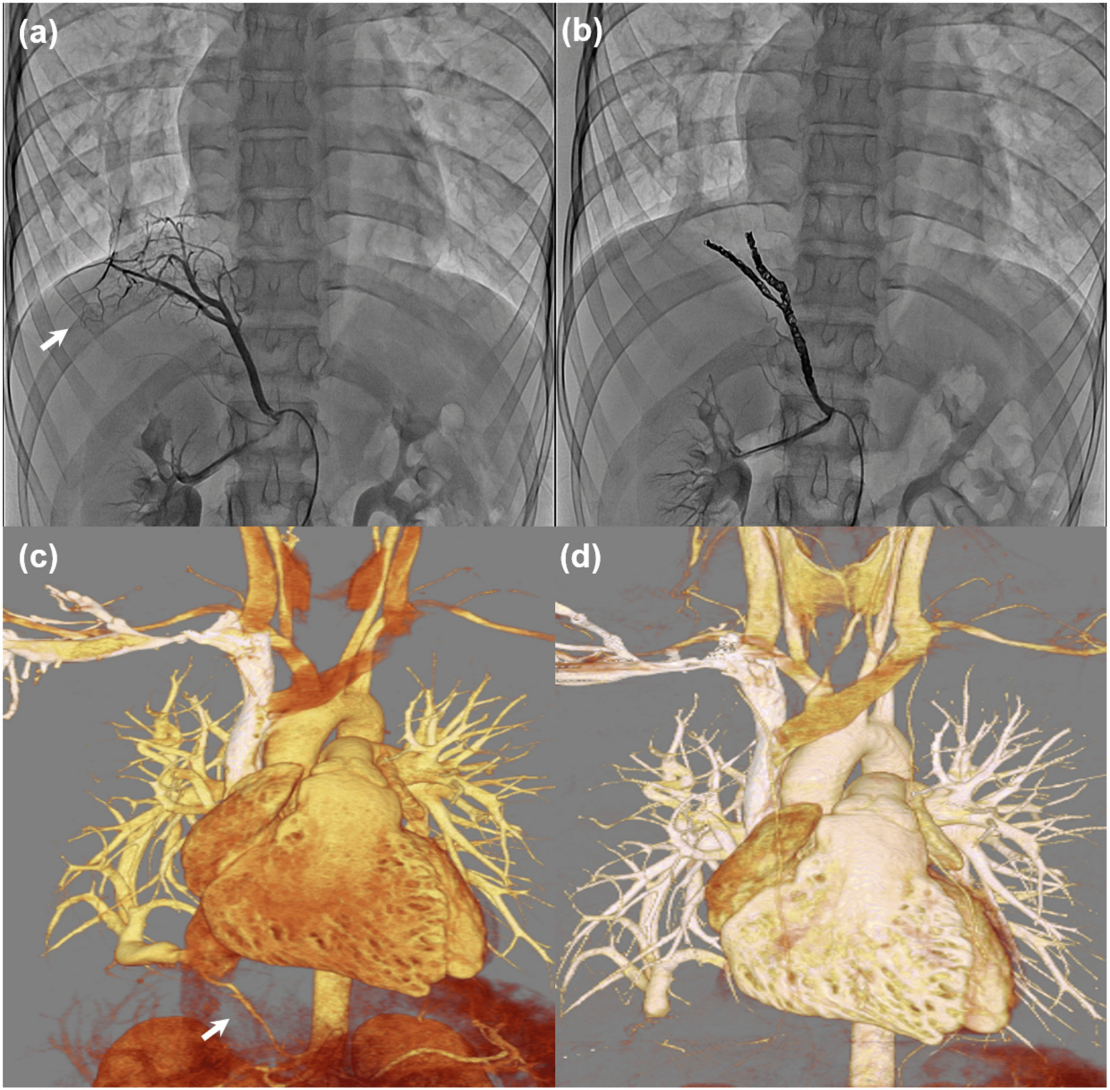
Angiography and 3D CT images of the patient at 19 years of age (a, b) Angiography images show an anomalous vessel originating from the right renal artery (a, arrow), which was blocked by coil embolization (b). (c, d) A 3D CT-based model of the patient's heart showed the scimitar vein (c, arrow), which is blocked off after the ligation (d).

At 19 years of age, she had recurrent pneumonia and hemoptysis in addition to her dyspnea on exertion (New York Heart Association (NYHA) II) [12]. Transesophageal echocardiography (TEE) revealed no atrial septal defect (ASD) but showed right heart enlargement. Furthermore, anomalous drainage of the right pulmonary vein into the inferior vena cava was observed. Cardiac catheterization denied pulmonary hypertension; her mean pulmonary arterial pressure was 15 mmHg (≥20 mmHg is considered elevated). Her modified WHO (mWHO) risk classification, the Zwangerschap bij Aangeboren HARtAfwijking (ZAHARA) score, and the CARdiac disease in PREGnancy (CARPREG) score were Class II, 0.75, and 1, respectively [13-15]. Since the anomalous vessel originating from the right renal artery was considered the cause of hemoptysis, coil embolization was performed (Figures 2a, 2b). Recanalization of the scimitar vein was not adopted because the scimitar vein was located more than 6.0 cm away from the left atrium. In addition, CT demonstrated collateral communication between the scimitar vein and the right inferior pulmonary vein, preserving right lung venous return to the left atrium. Therefore, video-assisted thoracoscopic ligation of the scimitar vein at its junction with the inferior vena cava was performed (Figures 2c, 2d). The postoperative course was uneventful, and she underwent annual follow-up examinations thereafter. Her dyspnea improved (NYHA I), and several echocardiography tests denied pulmonary hypertension. Her mWHO risk classification, ZAHARA score, and CARPREG score were class I, 0, and 0, respectively [12-15]. The cardiology team determined that pregnancy and delivery were permissible.

At 23 years of age, she conceived spontaneously and presented to our department at nine weeks of gestation for perinatal management. On physical examination, she was in good general condition, with a blood pressure of 113/65 mmHg. There were no obstetrical complications. Echocardiography was performed at 28 weeks of gestation, which indicated normal right ventricular systolic function (fractional area change (FAC) 44% (normal range: > 35%), tricuspid annular plane systolic excursion (TAPSE) 24.7 mm (normal range: > 17 mm)), and no significant pulmonary hypertension (the tricuspid regurgitation peak velocity (TRVp) of 2.0 m/s (normal range: < 2.5 m/s) with a trivial regurgitation, estimated right ventricle systolic pressure (RVSP) 19 mmHg). Therefore, routine prenatal care and standard delivery management were planned. She chose an epidural with labor induction per her preference, and she was admitted for cervical ripening at 39 weeks and 2 days of gestation; on physical examination and obstetrical assessments, no abnormalities were observed. On the following day (39 weeks and 3 days of gestation), her labor was induced with oxytocin, and epidural anesthesia was administered at her request. She achieved an uncomplicated vaginal delivery with a labor duration of 4 hours and 12 minutes and an estimated blood loss of 278 g. Her female newborn weighed 3090 g (appropriate for dates (AFD)) with Apgar scores of 8 and 9 at 1 and 5 minutes, and her umbilical artery pH (UApH) was 7.310. The newborn did not have any cardiac defects. After her postpartum period, she underwent cardiopulmonary exercise testing, which showed her peak oxygen consumption (VO_2_) was within normal range (24.3 ml/kg/min, 81% of predicted).

At 26 years of age, she conceived spontaneously and visited our department for perinatal management. At 10 weeks of gestation, echocardiography showed her cardiac function to be preserved (FAC 41%, TAPSE 28 mm, TRVp 1.4 m/s, RVSP 11 mmHg with trivial regurgitation). She did not have any dyspnea or abnormal weight gain. She did not have any restrictions on birth planning. She chose an epidural with labor induction, which resulted in an uncomplicated vaginal delivery at 38 weeks and 6 days with a labor duration of 5 hours and 45 minutes and an estimated blood loss of 156 g. Her female newborn weighed 3158 g (AFD) with Apgar scores of 8 and 9 at 1 and 5 minutes, and her UApH was 7.25. The newborn did not have any cardiac defects. On her latest visit, six months after her delivery, she had not developed any cardiopulmonary symptoms.

## Discussion

We report two uncomplicated vaginal deliveries in a woman with scimitar syndrome. To our knowledge, there have been eight deliveries in six women with scimitar syndrome (Table 1). Only two vaginal deliveries in two women have been reported; our patient was the third woman who experienced vaginal delivery. Furthermore, our patient was the first woman to experience vaginal deliveries after surgical treatment. This clinical experience adds to the limited literature on pregnancy management in scimitar syndrome and highlights the importance of management for patients with congenital heart defects in their adolescence and early adulthood, especially before pregnancy.

**Table 1:** Reported deliveries in women diagnosed with scimitar syndrome *C/S: caesarean section; PH: pulmonary hypertension; TR: tricuspid regurgitation

Reference	Age at diagnosis	Treatment history	Pulmonary pressure	Symptoms before pregnancy	Age at pregnancy	Obstetric complications	Patient's outcome	
							Weeks of delivery	Mode of delivery
Bell et al. [6]	Three months of age	Right pneumonectomy	Not available	Asthma	22 years	Dyspnea	37	C/S
					24 years	Dyspnea, oligohydramnios	33	C/S
Funata et al. [7]	Infancy	None	No PH at 29 years	Asthma	29 years	None	37	C/S
					32 years	None	38	C/S
Ohan et al. [8]	29 years	None	mild TR, No PH at 29 years	Asthma, mild TR	29 years	None	37	Labor analgesia
Hendrie et al. [9]	19 years	None	mild TR	Dyspnea on exertion	19 years	None	39	Labor analgesia
Yuksel et al. [10]	29 years	None	mild TR	None	29 years	Severe preeclampsia, right heart enlargement, mild tricuspid regurgitation	34	C/S
Althomali et al. [11]	33 years	None	Not available	Mild right heart enlargement, palpitations	33 years	Breech presentation	39	C/S
Present case	15 years	Ligation of the scimitar vein	15 mmHg (mean)	Dyspnea on exertion, pneumonia, hemoptysis	23 years	None	39	Labor analgesia
					26 years	None	38	Labor analgesia

Our patient was the first woman to experience vaginal deliveries after surgical treatment, which indicates that a woman with surgically palliated scimitar syndrome is capable of a vaginal delivery if her heart and lung functions are stable. The two other women who were reported to have scimitar syndrome and underwent vaginal deliveries did not undergo any surgical intervention before becoming pregnant, as their symptoms were sufficiently mild to preclude the necessity of surgery [8,9]. Even though she received surgical treatment for recurrent pneumonia, hemoptysis, and dyspnea upon exertion (NYHA II) [12], her post-surgical medical history was stable, and her perinatal condition was steady, mirroring the two reported patients. Surgical treatments were reported to mitigate the risk of late complications in patients. Our patient’s perinatal outcome underscores that surgical palliation can facilitate safe vaginal deliveries in women with scimitar syndrome.

Since symptoms in postpartum women with congenital heart defects can worsen months or even years after delivery [16], long-term follow-up is a critical necessity. Three years and six months passed between her first delivery and her latest examination, which denied the worsening of her cardiopulmonary symptoms. Other reported long-term follow-up records include two years and eight months and three years and two months; neither showed obvious worsening of symptoms [6,7]. In conjunction with our patient’s record, these available data indicate that pregnancy and childbirth do not affect scimitar syndrome, although additional clinical histories are required to reach a firm conclusion.

Due to the limited number of reported pregnancies, establishing a standard procedure for risk assessment and determining contraindications for natural deliveries in scimitar syndrome patients is challenging. For women prior to their childbearing age, a preconceptional strategy, including careful counseling, is critical because surgically uncorrected scimitar syndrome could become exacerbated with age and has the potential risk of complications during pregnancy, predominantly in those with elevated pulmonary pressures or recurrent pulmonary infections.

## Conclusions

Based on our experience and literature review, in patients with scimitar syndrome who have normal pulmonary pressures and no ongoing respiratory infections, irrespective of prior interventions, the risk of pregnancy-related complications appears to be low. To date, no cases describing adverse pregnancy outcomes in this clinical context have been reported. More cases are necessary to delineate pregnancy outcomes in women with scimitar syndrome, which enables a better multidisciplinary approach, preconceptional counseling, and the establishment of the management protocol during and after their pregnancies.
